# Investigation of Genetic Variations of *IL6* and *IL6R* as Potential Prognostic and Pharmacogenetics Biomarkers: Implications for COVID-19 and Neuroinflammatory Disorders

**DOI:** 10.3390/life10120351

**Published:** 2020-12-16

**Authors:** Claudia Strafella, Valerio Caputo, Andrea Termine, Shila Barati, Carlo Caltagirone, Emiliano Giardina, Raffaella Cascella

**Affiliations:** 1 Genomic Medicine Laboratory, IRCCS Santa Lucia Foundation, 00179 Rome, Italy; valerio.caputo@alumni.uniroma2.eu (V.C.); andrea.termine.02@students.uniroma2.eu (A.T.); s.barati@hsantalucia.it (S.B.); emiliano.giardina@uniroma2.it (E.G.); raffaella.cascella@uniroma2.it (R.C.); 2 Department of Biomedicine and Prevention, Tor Vergata University, 00133 Rome, Italy; 3 Department of Clinical and Behavioral Neurology, IRCCS Fondazione Santa Lucia, 00179 Rome, Italy; c.caltagirone@hsantalucia.it; 4 UILDM Lazio ONLUS Foundation, Department of Biomedicine and Prevention, Tor Vergata University, 00133 Rome, Italy; 5 Department of Biomedical Sciences, Catholic University Our Lady of Good Counsel, 1000 Tirana, Albania

**Keywords:** biomarkers, IL6, IL6R, SARS-Cov-2, COVID-19, neurodegenerative disorders, genetic variants, personalized medicine

## Abstract

In the present study, we investigated the distribution of genetic variations in *IL6* and *IL6R* genes, which may be employed as prognostic and pharmacogenetic biomarkers for COVID-19 and neurodegenerative diseases. The study was performed on 271 samples representative of the Italian general population and identified seven variants (rs140764737, rs142164099, rs2069849, rs142759801, rs190436077, rs148171375, rs13306435) in *IL6* and five variants (rs2228144, rs2229237, rs2228145, rs28730735, rs143810642) within *IL6R*, respectively. These variants have been predicted to affect the expression and binding ability of IL6 and IL6R. Ingenuity Pathway Analysis (IPA) showed that IL6 and IL6R appeared to be implicated in several pathogenetic mechanisms associated with COVID-19 severity and mortality as well as with neurodegenerative diseases mediated by neuroinflammation. Thus, the availability of *IL6*-*IL6R*-related biomarkers for COVID-19 may be helpful to counteract harmful complications and prevent multiorgan failure. At the same time, *IL6*-*IL6R*-related biomarkers could also be useful for assessing the susceptibility and progression of neuroinflammatory disorders and undertake the most suitable treatment strategies to improve patients’ prognosis and quality of life. In conclusion, this study showed how IL6 pleiotropic activity could be exploited to meet different clinical needs and realize personalized medicine protocols for chronic, age-related and modern public health emergencies.

## 1. Introduction

**Figure 1:**
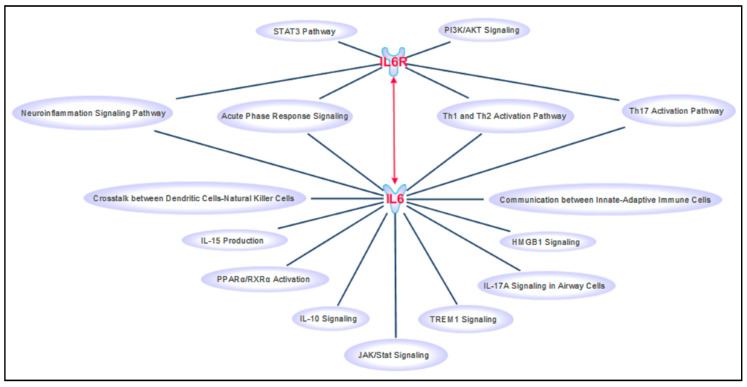
Canonical pathways involving IL6 and IL6R retrieved by IPA software. This figure has been created by “Path Designer” IPA tool.

The identification of biological markers (i.e., biomarkers) of disease detectable in several biological fluids and tissues represents the key milestone for the implementation of personalized medicine protocols into the clinical practice [1,2,3,4,5]. The need of clinically useful biomarkers and personalized medicine strategies became even more important with the recent outbreak of the novel pathogenic severe acute respiratory syndrome coronavirus 2 (SARS-Cov-2) and the resulting coronavirus infectious disease (COVID-19) pandemics [6,7]. However, COVID-19 is just the most recent challenge for the healthcare system, which has also to tackle with a growing aging population and the diverse pathological conditions affecting the elderly, such as neurodegenerative disorders. Interestingly, neurodegenerative disorders and viral infection pathologies share several molecular signatures which, altogether, indicate the dysfunction of the local and peripheral immune system response as a common etiopathogenetic mechanism [8,9,10]. In this context, the availability of specific biomarkers could be extremely helpful to identify at-risk individuals, select the most suitable therapy and counteract the progression and mortality of such public health emergencies. Two of the most promising candidate biomarkers for both COVID-19 and neurodegenerative disorders are interleukin-6 (IL6) and its receptor (IL6R). IL6 consists of a four-helix bundle conformation and exerts its functions by binding the interlukin-6 receptor (IL6R) [11]. IL6R can be either membrane-bound (classical signaling) on the extracellular surface of immune, epithelial and liver cells, or it can be circulating in soluble form (trans-signaling pathway) and act as IL6 agonist [12,13]. The IL6-IL6R complex interacts with the glycoprotein 130 (gp130) membrane receptor which, in turn, triggers downstream intracellular signaling pathways (Figure 1) mainly involved in the immunoinflammatory response [11,13].

Genetic variants in *IL6* (7p15.3) and *IL6R* (1q21.3) genes have been supposed to affect the binding ability, expression levels and biological functions of the IL6-IL6R complex, contributing thereby to the onset and progression of severe infectious, autoimmune and neuroinflammatory/neurodegenerative diseases, including COVID-19, hepatitis B infection, rheumatoid arthritis (RA), cardiovascular disorders (CVD), multiple sclerosis (MS), Alzheimer’s disease (AD) and Parkinson’s disease (PD) [14,15,16,17]. Indeed, a number of studies identified variants located in the regulatory regions of *IL6* and *IL6R* as genetic determinants of high IL6 circulating levels in serum and tissues that have been proposed to affect the risk and progression of many different disease states (especially COVID-19, CVD and AD) [16,17,18]. In the present study, we investigated the distribution of copy number variations (CNVs) and genetic variants located within the coding sequences of *IL6* and *IL6R* genes, which may be employed as prognostic and drug response (pharmacogenetic) biomarkers for COVID-19 and neuroinflammatory diseases. We decided to focus the attention on these pathologies because they both represent current public health issues and the identification of biomarkers within *IL6* and *IL6R* could provide therapeutic strategies relevant to both pathological conditions.

## 2. Materials and Methods

The study was performed on a cohort of 271 DNA samples representative of the Italian general population. The study cohort was composed of 100 samples analyzed by array comparative genomic hybridization (aCGH) for assessing the presence of structural genomic variations and 171 samples utilized for identifying common and rare single nucleotide variants (SNVs) located in the coding or splice site regions of the genome. Genetic data were partially obtained from aCGH and whole exome sequencing (WES) data available at the Genomic Medicine Laboratory of IRCCS Santa Lucia Foundation and partially retrieved from Ensembl [19,20,21]. The use of laboratory data for research purposes was approved by the Ethics Committee of IRCCS Santa Lucia Foundation of Rome (CE/PROG.650 approved on 1 March 2018) and by the signed informed consent provided by the individuals subjected to genetic testing at our laboratory.

The CNV analysis was performed by Chromosome Analysis Suite (ChAS) 3.1 (Affymetrix, Santa Clara, CA, USA) using the Cytoscan750k_Array Single Sample analysis: NA33_hg19 as reference file and an average resolution of 100 kb. Concerning SNVs, Ensembl [19], 1000Genomes [20] and GnomAD [21] databases were utilized to extract the frequency data of the exonic variants of interest. In particular, 22 variants located within IL6 and 37 variants within IL6R were selected. Successively, the presence and the frequency distribution of the selected variants have been evaluated in the cohort of Italian samples. For WES results, a coverage of 20X was considered for the analysis of the IL6 and IL6R sequence. The variant caller files (VCF) obtained by WES analysis were firstly scanned with vcfR and then subjected to “genomic variants filtering by deep learning models in NGS” (GARFIELD-NGS) analysis. In particular, vcfR is a package that enables to visualize, manipulate and perform the quality control of VCF data [22]. GARFIELD-NGS is an informatics tool which relies on deep learning models to dissect false and true variants in exome sequencing experiments [23]. Concerning the evaluation of frequency distributions of the selected variants, minor allele frequency (defined as the less common allele of the SNVs of interest) was taken into account to compare their frequencies in the Italian population.

**Figure 2:**
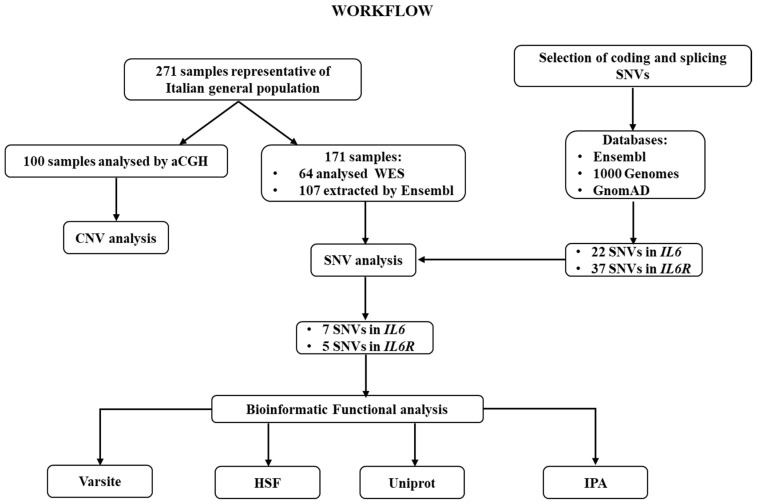
Workflow of the analytical steps performed in the study. CNV: Copy Number Variation; SNV: Single Nucleotide Variants; HSF: Human Splicing Finder; IPA: Ingenuity Pathway Analysis.

Successively, the identified variants were subjected to bioinformatic predictive analysis in order to assess their potential impact on protein expression and function. In particular, VarSite and Human Splicing Finder (HSF) were interrogated. VarSite analyzes and predicts the effect of amino acid changes on the protein structure [24]. HSF evaluates the effects of variants on the splicing mechanisms [25]. Moreover, the Uniprot annotation database was utilized to retrieve the topological and functional domains organization of proteins [26]. Moreover, Ingenuity Pathway Analysis (IPA, Qiagen) software application was performed in order to place IL6 and IL6R into their biological context and postulate their possible association with COVID-19 severity and neuroinflammatory disorders and their potential use as druggable targets. IPA is an all-in-one web-based software application that allows the analysis and integration of different kinds of genetic data, facilitating their interpretation, the identification of specific targets or candidate biomarkers and placing them in the context of larger biological or chemical systems. The software is backed by the Ingenuity Knowledge Base, which consists of highly structured, detail-rich biological and chemical findings. The entire analytical workflow of the study has been illustrated in Figure 2.

## 3. Results and Discussion

The present study investigated the distribution of CNVs and SNVs located within the coding sequences of *IL6* and *IL6R* genes, with the aim of identifying candidate prognostic and pharmacogenetic biomarkers for COVID-19 and neuroinflammatory diseases. The analysis of CNVs did not report any significant variation in our study cohort ruling out that frequent copy number variations could potentially impact *IL6* and *IL6R* expression. Concerning SNVs instead, 22 variants located within *IL6* and 37 variants within *IL6R* were selected and investigated in the cohort of Italian samples. As a result, seven variants located within *IL6* and five variants within *IL6R* were identified, respectively (Table 1). Among the variants of *IL6*, three were synonymous (rs140764737, C/T; rs142164099, G/A, rs2069849, C/T) and four missense (rs142759801, C/A; rs190436077, G/C; rs148171375, A/T and rs13306435, T/A). These variants appeared to be rare in the Italian cohort, with minor allele frequency (MAF) ranging from 0.009 to 0.003 (Table 1).

Concerning the synonymous variants, only rs140764737 has been predicted to impact the regulation of splicing mechanisms. According to HSF, the T variant allele may disrupt an exonic splicing enhancer (ESE) site and create a new exonic splicing silencer (ESS). The analyses conducted on the missense variants unveiled that rs142759801 (C/A) may affect *IL6* expression and function through the impairment of canonical splicing mechanisms. In fact, HSF interrogation reported a potential alteration of an ESE site. VarSite did not report any effect on protein structure. Furthermore, bioinformatic predictive analysis suggested that rs190436077 and rs13306435 may impact protein structure because of their amino acid substitution, whereas rs148171375 was not predicted to affect protein function, structure and splicing activity. In fact, the rs190436077 causes a glutamate (with a negatively charged side chain) to glutamine (carrying a neutral side chain) change at the 79th amino acid and it has also been predicted to disrupt an ESE site. Moreover, it falls within the loop connecting the first two helical structures of the protein, which contributes to the formation of the binding site for IL6/IL6R complex to gp130 (23). The rs190436077 may therefore be experimentally investigated to verify its potential role on the alteration of IL6 binding ability and could be also evaluated for potential effects on the affinity with IL6 drugs, which may cause an altered drug response or effectiveness. Concerning the variants located within *IL6R*, two synonymous (rs2228144, G/A and rs2229237, C/T) and three missense variants (rs2228145, A/C; rs28730735, C/T and rs143810642, C/T) were detected in the Italian cohort. These variants showed variable frequency distributions, with the rs2228144 (MAF: 0.178) and rs2228145 (MAF: 0.327) being the most frequently observed (Table 1). Concerning the synonymous variants, bioinformatic analysis supported an effect on the splicing mechanisms for the rs2229237 variant, which was predicted to activate a cryptic acceptor site and alter the regulatory splicing sequences (Table 1). All the missense variants were predicted to alter ESE sites and/or create new ESS sites, whereas the rs2228145 was also predicted to impact the protein function due to the amino acid substitution from an aspartate (Asp, with a negatively charged side chain) to alanine (Ala, with an aliphatic side chain) at the 358th residue. Interestingly, this amino acid variant is located within the extracellular domain of the receptor, which is fundamental for IL6R interaction with extracellular ligands. Therefore, the variant may alter the domain conformation, potentially interfering with IL6 recognition. Indeed, the C allele of rs2228145 is strongly associated with increased levels of soluble IL6R in blood, serum and cerebrospinal fluid (CSF) [11,27]. This finding may be explained by the fact that the Ala residue makes the conformation at this site more susceptible to the cleavage, leading to increased levels of soluble IL6R [11]. Although rs2228145 has been described as a major determinant of circulating soluble IL6R levels, its effect on IL6 signaling and inflammatory response remains unclear. Some studies reported that the variant allele of the rs2228145 may modulate the balance with membrane-bound and soluble IL6R, affect the responsiveness of immune cells to IL6 stimulation and may also affect disease onset progression in some cases [28,29,30,31]. On the other hand, another work found a significant change in serum levels of IL6R and, to a lesser extent, of IL6 levels, but did not show any association with gp130 levels or pro/anti-inflammatory markers tested [32]. Therefore, the functional role of rs2228145 in the inflammatory status and its possible effects within tissue-specific contexts are still under debate and need further investigation. Nevertheless, these findings encourage the exploration of the functional genetic variation in *IL6R* for identifying pharmacogenetic biomarkers or applying personalized/stratified medicine approaches in people with different genetic profiles.

**Figure 3:**
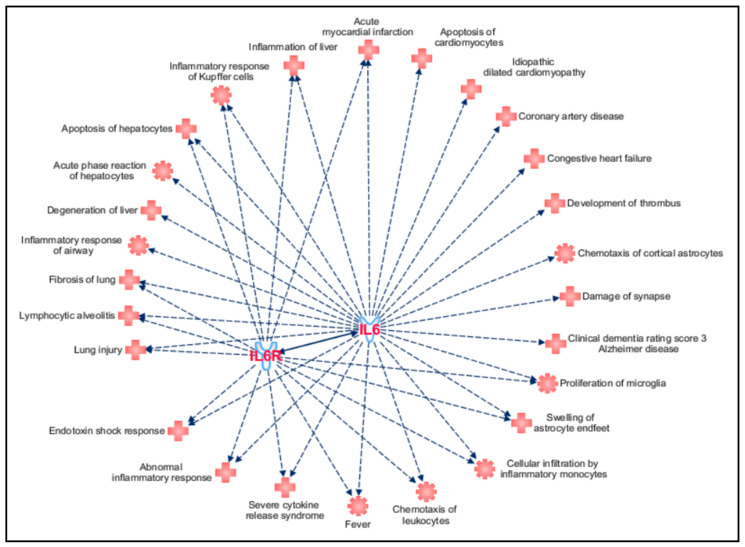
Pathophysiological conditions in which IL6 and IL6R may be implicated following the “Disease & Functions” analysis performed on IPA tool. The figure has been created by the “Path Designer” IPA tool.

Over the investigation of coding variants in *IL6* and *IL6R* genes, we performed a “Disease and Function” analysis on IPA to visualize the pathophysiological pathways in which IL6 and IL6R may be implicated, how they could affect the severity/progression of COVID-19 and neuroinflammatory disease and their possible use as druggable targets for these conditions. According to this analysis, IL6 and IL6R appeared to be implicated in several pathogenetic mechanisms associated with COVID-19 severity and mortality, especially affecting lungs, liver, heart and nervous system (Figure 3).

Notably, the lung is the most affected organ by SARS-Cov-2, whose infection triggers acute immunoinflammatory responses culminating in decreased oxygen uptake, lung injury and severe pneumonia [33]. Moreover, acute cardiac injury (arrhythmias, myocardial infarction and heart failure) and abnormal blood clotting have been reported as complications of SARS-Cov-2 infection in approximately 20–30% and 38% of COVID-19 patients, respectively [33,34]. Cardiac and blood vessel involvement can result by direct and indirect mechanisms, including viral infiltration into myocardial tissue (causing cardiomyocyte death and inflammation), stress induced by respiratory failure and hypoxemia and inflammation due to severe systemic hyperinflammation [35]. In 14–53% cases, abnormal levels of alanine aminotransferase, aspartate aminotransferase, lactate dehydrogenase, lymphopenia have been associated with hepatic dysfunction and liver injury [35,36]. These alterations may be either a consequence of direct viral invasion or may be due to drug hepatotoxicity and immune system overdrive. In addition, 14–36% of severe COVID-19 patients reported neurological symptoms such as taste and smell impairment, dizziness, seizures, impaired consciousness, encephalitis and stroke [37,38]. Even in this case, neurological symptoms could depend on brain viral infection or on the systemic hyperinflammation and abnormal blood clotting. 

Considering that most severe COVID-19 cases display neurological symptoms, the need of specific biomarkers is extremely important to identify at-risk patients, provide timely treatments and closer monitoring as well as improve prognosis and long-term neurological outcomes of disease [6,7,39]. Although no specific biomarkers have been linked to neurological manifestations in COVID-19 patients up to date, two studies found correlations between IL6 levels and taste/smell dysfunctions and headache, respectively [40,41]. In addition, increased IL6 levels (together with IL8 and IP10) have been found in the CSF of one patient affected with COVID-19 and displaying an altered mental status and evidence of seizures [42]. Interestingly, the patient reported elevated inflammation in CSF, although the CSF was negative to SARS-Cov-2 virus. This finding suggests that neurological symptoms may be due to increased neuroinflammation rather than CNS invasion of virus [42]. This case report highlights that COVID-19 may present with acute neurological symptoms (such as encephalopathy and seizures) in association with elevated inflammatory markers in CSF, in the absence of respiratory illness [39,42]. However, such hypothesis deserves further investigation and should be followed up in other cases of seizure and encephalopathy during COVID-19. Although in its infancy, research on IL6 and IL6R could be extremely helpful to identify biomarkers relevant to COVID-19-related neuroinflammation and set up personalized treatment and monitoring strategies aimed at avoiding dangerous long-term neurological outcomes of COVID-19.

Over the association of IL6 and IL6R with COVID-19 and complications, the “Disease and Function” analysis reported that IL6 and IL6R were also implicated in damage of synapses, microglia proliferation, astrocytes swelling and severe dementia in AD (namely, clinical dementia rating score 3 Alzheimer’s disease) (Figure 3). These data, together with the evidence of association between high IL6 levels and neuroinflammation [11], advocate for a role of IL6 and IL6R as molecular contributors to AD progression and designate them as candidate druggable targets for AD and other neurodegenerative diseases mediated by neuroinflammation.

Considering the above-presented data, we encourage similar studies on other populations to verify the existence of population-specific genomic profiles, which could contribute to the differential susceptibility and progression of COVID-19 and/or neuroinflammatory diseases as well as to the variable drug response. Human and animal studies showed that IL6 and IL6R are excellent targets for immunomodulatory therapies and could be used as biomarkers of disease activity because of their pleiotropic effects in several tissues (liver, brain, bone, lung, skeletal muscle, heart) and biological fluids (blood, serum/plasma, urine) [7,43,44,45,46,47]. Several drugs (sirukumab, clazakizumab, siltuximab and olokizumab) targeting IL6 have been designed and are currently approved or under investigation for treating RA, Chron’s disease, depression, Lupus nephritis and Castleman disease. Concerning IL6R-targeting drugs, sarilumab is currently indicated for moderate to severe active RA, whereas tocilizumab is utilized in the treatment of moderate to severe RA, giant cell arteritis, polyarticular juvenile idiopathic arthritis, systemic juvenile idiopathic arthritis and cytokine release syndrome. In addition, tocilizumab is currently under investigation as a treatment option for patients affected with severe COVID-19 [48,49]. Given this data, the availability of *IL6*-*IL6R*-related biomarkers for COVID-19 may be helpful to counteract or timely treat harmful complications and prevent multiorgan failure. At the same time, *IL6*-*IL6R*-related biomarkers could also be useful for assessing the susceptibility and progression of neuroinflammatory disorders and undertake the most suitable treatment strategies in order to improve patients’ prognosis and quality of life. In conclusion, this study showed how IL6 pleiotropic activity could be exploited to meet different clinical needs and achieve the realization of personalized medicine protocols for chronic, age-related and modern public health emergencies.

## Figures and Tables

**Table 1:** Exonic variants located within *IL6* and *IL6R* found in the cohort of 171 samples representative of the Italian general population. The table reports the gene and genomic location of each variant according to GRCh37 release, SNV, nucleotide and protein coding, allele counts (frequencies) in the study cohort, exonic location and the impact of variants on structure (VarSite) and splicing (HSF). SNV: Single Nucleotide Variant. N coding: nucleotide coding. Ex: Exon; P coding: Protein coding. AA: amino acid. Minor allele frequency (MAF) has been labelled with an asterisk (*).

Gene	Genomic Location	SNV	N Cod	Allele Counts (Frequencies)	Ex	P Cod	Structural Impact of AA Change (Varsite)	Impact on Splicing (HSF)
*IL6* (7p15.3)	7:22767134	rs142759801	c.91 C > A	C: 341 (0.997) A: 1 (0.003) *	2	p.P31T	low impact on protein structure	alteration of an ESE site
	7:22767226	rs140764737	c.183 C > T	C: 341 (0.997) T: 1 (0.003) *	2	p.L61=	NA	alteration of an ESE site, creation of an ESS site
	7:22768336	rs190436077	c.235 G > C	G: 341 (0.997) C: 1 (0.003) *	3	p.E79Q	potential impact on protein structure	alteration of an ESE site
	7:22768350	rs142164099	c.249 G > A	G: 341 (0.997) A: 1 (0.003) *	3	p.E83=	NA	no predicted impact
	7:22769154	rs148171375	c.346 A > T	A: 341 (0.997) T: 1 (0.003) *	4	p.I116F	low impact on protein structure	no predicted impact
	7:22771039	rs13306435	c.486 T > A	T: 339 (0.991) A: 3 (0.009) *	5	p.D162E	potential impact on protein structure	no predicted impact
	7:22771156	rs2069849	c.603 C > T	C: 339 (0.991) A: 3 (0.009) *	5	p.F201=	NA	no predicted impact
*IL6R* (1q21.3)	1:154401679	rs2228144	c.93 G > A	G: 281 (0.822) A: 61 (0.178) *	2	p.A31=	NA	no predicted impact
	1:154401796	rs2229237	c.210 C > T	C: 338 (0.988) T: 4 (0.012) *	2	p.H70=	NA	alteration of an ESE site, creation of an ESS site
	1:154426970	rs2228145	c.1073 A > C	A: 230 (0.673) C: 112 (0.327) *	9	p.D358A	potential impact on protein structure	alteration of an ESE site, creation of an ESS site
	1:154427032	rs28730735	c.1135 C > T	C: 340 (0.994) T: 2 (0.006) *	9	p.L379F	low impact on protein structure	alteration of an ESE site, creation of an ESS site
	1:154437719	rs143810642	c.1270 C > T	C: 340 (0.994) T: 2 (0.006) *	10	p.L424F	low impact on protein structure	alteration of an ESE site, creation of an ESS site
